# Transient receptor potential ankyrin 1 contributes to somatic pain hypersensitivity in experimental colitis

**DOI:** 10.1038/s41598-020-65618-5

**Published:** 2020-05-25

**Authors:** Piyush Jain, Serena Materazzi, Francesco De Logu, Duccio Rossi Degl’Innocenti, Camilla Fusi, Simone Li Puma, Ilaria M. Marone, Elisabetta Coppi, Peter Holzer, Pierangelo Geppetti, Romina Nassini

**Affiliations:** 1 Section of Clinical Pharmacology and Oncology, Department of Health Sciences, University of Florence, Florence, Italy; 2 Research Unit of Translational Neurogastroenterology, Division of Pharmacology, Otto Loewi Research Center, Medical University of Graz, Graz, Austria; 3 EMOLED S.r.L., Sesto Fiorentino, Florence, Italy; 4 Research Group Neuroplasticity, Leibniz Institute for Neurobiology, Magdeburg, Germany; 5 Department of Neuroscience, Psychology, Drug Research and Child Health, Section of Pharmacology and Toxicology, University of Florence, Florence, Italy

**Keywords:** Inflammatory bowel disease, Digestive signs and symptoms, Pain

## Abstract

Pain evoked by visceral inflammation is often ‘referred’ to the somatic level. Transient receptor potential ankyrin 1 (TRPA1) has been reported to contribute to visceral pain-like behavior in dextran sulfate sodium (DSS)-evoked colitis. However, the role of TRPA1 in somatic component of hypersensitivity due to visceral inflammation is unknown. The present study investigated the role of TRPA1 in colitis-evoked mechanical hypersensitivity at the somatic level. Colitis was induced in mice by adding DSS to drinking water for one week. Control and DSS-treated mice were tested for various parameters of colitis as well as mechanical pain sensitivity in abdominal and facial regions. DSS treatment caused mechanical hypersensitivity in the abdominal and facial skin. Pharmacological blockade or genetic deletion of TRPA1 prevented the colitis-associated mechanical hypersensitivity in the abdominal and facial skin areas although the severity of colitis remained unaltered. DSS treatment increased expression of TRPA1 mRNA in cultured dorsal root ganglion (DRG) neurons, but not trigeminal ganglion neurons, and selectively enhanced currents evoked by the TRPA1 agonist, allyl isothiocyanate, in cultured DRG neurons. Our findings indicate that the TRPA1 channel contributes to colitis-associated mechanical hypersensitivity in somatic tissues, an effect associated with upregulation of TRPA1 expression and responsiveness in DRG nociceptors.

## Introduction

Inflammatory bowel disease (IBD) encompasses two principal types of chronic inflammation in the intestine, ulcerative colitis (UC) and Crohn’s disease (CD), which share several common pathophysiologic features (disturbances of immune system, mucosal barrier function and gut microbiota) and symptoms (abdominal pain, presence of blood in stool, diarrhea and weight loss)^1,2^. Although usually not life-threatening, IBD severely affects patient’s quality of life especially because of the presence of chronic pain^3^^,^^4^, which is poorly amenable to pharmacologic treatment^5^. Thus, a better understanding of the underlying mechanisms of abdominal pain associated with IBD is crucial to identify novel targets for pharmacologic intervention. Although visceral pain originates in internal organs, abdominal pain is often referred to the somatic level, because nociceptive afferent fibers supplying the abdominal viscera converge with somatic nociceptive afferents in the dorsal horn of the spinal cord^6^. For this reason, referred pain can be used as an experimental measure of intestinal pain^7,8^. Referred pain is also observed in UC and CD patients^5,9^ and IBD can be associated with a variety of other extraintestinal manifestations^10^. For instance, there is a significant association of migraine with IBD^11^, and treatment of gastrointestinal (GI) disorders has been found to improve headache/migraine symptoms^12–14^.

The transient receptor potential ankyrin 1 (TRPA1) channel, belonging to the larger family of TRP channels, is activated by chemical and mechanical stimuli. In mammals, TRPA1 is abundantly expressed in a subpopulation of dorsal root ganglion (DRG) and trigeminal ganglion (TG) primary sensory neurons which subserve nociceptive functions^15,16^. In addition, these neurons express and release neuropeptides such as substance P (SP) and calcitonin gene-related peptide (CGRP) that mediate neurogenic inflammatory processes^15,16^.

Apart from exogenous compounds such as allyl isothiocyanate (AITC) and cinnamaldehyde, a range of endogenous byproducts of oxidative stress is able to selectively gate TRPA1, thus causing neuronal activation/sensitization^17–21^. In this capacity, TRPA1 has been implicated in models of inflammatory and neuropathic pain^16^. This also applies to the intestine in which TRPA1 appears to mediate mechanical hypersensitivity to colonic distension in chemically induced colitis^22,23^. Stimulation of TRPA1 by colorectal instillation of the TRPA1 agonist AITC elicits afferent input to the spinal cord and brain as visualized by activation of c-Fos and mitogen-activated protein (MAP) kinases and induces pain-like behaviors^24,25^.

Dextran sulfate sodium (DSS) dissolved in the drinking water is used to induce experimental inflammation of the colon and to study IBD-like conditions in rodents^26^. DSS-induced colitis reproduces several pathophysiologic features of UC such as increased expression of interleukin (IL)-6, IL-10 and tumor necrosis factor-α, elevated colonic levels of myeloperoxidase (MPO), inflammatory histopathology, bloody diarrhea and a decrease of ingestion^27,28^. Furthermore DSS-induced colitis is associated with sensitization of TRPA1 in the colon, which through release of substance P contributes to induction and maintenance of inflammation^29^. An implication of TRPA1 is evident from the observation that DSS-induced colitis is reduced by genetic deletion and pharmacologic blockade of TRPA1^29^. GI inflammation evoked by DSS is associated not only with intestinal hyperalgesia but also with mechanical hypersensitivity of the abdominal skin^24,26^_._ This phenomenon of referred intestinal pain in rodents^7,8,24^ can be evaluated with the von Frey hair test on the abdominal surface, a mechanical nociceptive threshold test that is also used to measure mechanical hypersensitivity in humans^30^. However, the contribution of TRPA1 to the refereed somatic pain that originates from inflamed visceral structure is unknown.

In our previous study^24^ we observed mechanical and thermal hypersensitivity (measured by the von Frey hair test and plantar test, respectively) in the hind paw of mice with DSS-induced colitis. Since a contribution of TRPA1 to referred somatic pain that originates from inflamed visceral structures is not yet known, by pharmacological and genetic tool we explored a potential role of TRPA1 in the colitis-evoked referred hypersensitivity in the abdominal and periorbital skin areas. Two specific hypotheses were addressed: (1) TRPA1 is involved in the somatic mechanical hypersensitivity associated with DSS-induced colitis. To this end, we assessed colonic inflammation and recorded the mechanical hypersensitivity of the abdominal and periorbital skin in control and DSS-treated mice after administration of a TRPA1 antagonist and in mice with TRPA1 genetic deletion. (2) The somatic mechanical hypersensitivity of DSS-treated mice is associated with altered expression of TRPA1 mRNA in the DRG and TG and altered sensitivity of DRG and TG cells to a TRPA1 agonist.

## Results

### Pharmacological TRPA1 blockade and genetic TRPA1 deletion reduced colitis-evoked somatic hypersensitivity to mechanical stimuli

**Figure 1:**
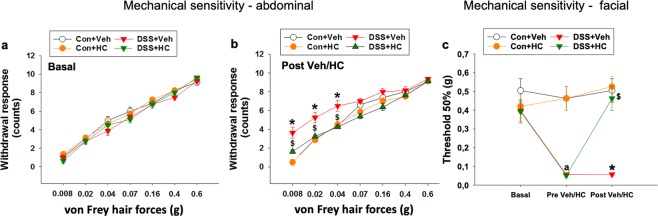
Effect of HC-030031 (HC) or its vehicle (Veh) in control (Con) mice and mice treated with DSS for 7 days on mechanical pain sensitivity of the abdominal (**a,b**) and periorbital (facial, **c**) region measured on day 0 (basal conditions, **a**) and day 8 (**b,c**). HC (100 mg/kg, i.p.) or its vehicle was injected one hour prior to the test. Values are expressed as means +/− SEM (n = 8). Repeated measures ANOVA did not show any DSS × HC × forces interaction in the withdrawal responses to abdominal stimulation under basal conditions (**a**). Under post Veh/HC conditions (**b**), repeated measures ANOVA demonstrated a DSS × HC × forces interaction at the 0.008, 0.02 (*P* < 0.01) and 0.04 g (*P* < 0.001) test forces. Bonferroni’s multiple comparison analysis revealed that the withdrawal responses to forces of 0.008 g (*P* < 0.001 vs. Con + Veh), 0.02 g (*P* < 0.001 vs. Con + Veh) and 0.04 g (*P* < 0.001 vs. Con + Veh) were significantly increased in DSS-treated mice (**b**). HC-030031 administration to DSS-treated mice led to a significant decrease in the withdrawal response to forces of 0.008 g (*P* < 0.01 vs. DSS + Veh), 0.02 g (*P* < 0.001 vs. DSS + Veh) and 0.04 g (*P* < 0.001 vs. DSS + Veh). In the periorbital (facial) region, there was no DSS × HC interaction and any DSS or HC effect on the withdrawal threshold to mechanical stimulation under basal conditions (two-way ANOVA, **c**). Two-way ANOVA of the data obtained under pre Veh/HC conditions revealed a DSS effect (^a^*P* < 0.001, c), and post-hoc Bonferroni’s multiple comparison analysis demonstrated significant differences under post Veh/HC conditions: *P < 0.05 vs. Con + Veh, ^$^*P* < 0.05 vs. DSS + Veh (**c**).

DSS treatment induced mechanical hypersensitivity in the mouse abdominal and periorbital regions (Fig. 1a–c). In order to explore the contribution of TRPA1 to this colitis-related hypersensitivity, DSS-treated mice received the selective TRPA1 antagonist HC-030031 or its vehicle. While the abdominal withdrawal response under basal conditions (prior to any treatment) did not differ between the experimental groups (Fig. 1a), the withdrawal response measured after DSS treatment prior to HC-030031/vehicle administration was significantly enhanced relative to that measured in control animals (Fig. 1b). This abdominal mechanical hypersensitivity was reduced by i.p. injection of HC-030031 (*P* < 0.01 by two-way ANOVA, Fig. 1b). Two-way ANOVA disclosed a significant interaction between DSS and HC-030031 at the test forces of 0.008 g, 0.02 g and 0.04 g, and post-hoc testing with Bonferroni’s multiple comparison analysis revealed that the withdrawal responses to these test forces were significantly increased in DSS-treated mice while HC-030031 administration to DSS-treated mice reversed the hypersensitivity to these test forces (Fig. 1b).

Under basal conditions there was no difference between the experimental groups in their withdrawal threshold to mechanical stimulation of the periorbital region (Fig. 1c). DSS treatment for 7 days lowered the withdrawal threshold to a significant extent (Fig. 1c). This colitis-related mechanical hypersensitivity of the facial region was attenuated by HC-030031 (*P* < 0.01 by two-way ANOVA, Fig. 1c). Taken together, these data show that DSS-induced colitis caused mechanical hypersensitivity in the abdominal and periorbital region and that blockade of TRPA1 with HC-030031 reversed the DSS-induced mechanical hypersensitivity in both regions.

**Figure 2:**
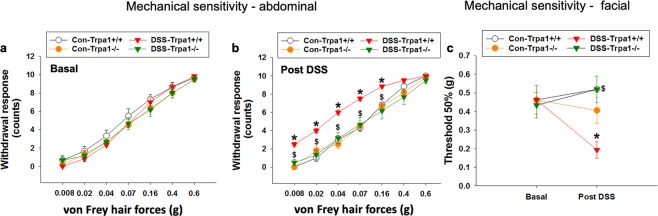
Effect of genetic deletion of TRPA1 in control (Con) mice and mice treated with DSS for 7 days on mechanical pain sensitivity of the abdomen (**a,b**) and periorbital (facial, **c**) region measured on day 0 (basal conditions, **a**) and day 8 (**b,c**). Values are expressed as means +/− SEM (n = 6). Repeated measures ANOVA did not show any DSS × genotype × forces interaction in the withdrawal response to abdominal stimulation under basal conditions (**a**). Following control or DSS treatment (post DSS, b), repeated measures ANOVA demonstrated a DSS × genotype × forces interaction at the 0.008 g (*P* < 0.001), 0.02 g (*P* < 0.01), 0.04 g (*P* < 0.05), 0.07 g and 0.16 g (*P* < 0.001) test forces. Bonferroni’s multiple comparison analysis revealed that the withdrawal responses to forces of 0.008 g, 0.02 g, 0.04 g, 0.07 g (*P* < 0.001 vs. Con-*Trpa1*^+/+^) and 0.16 g (*P* < 0.05 vs. Con-*Trpa1*^+/+^) were significantly increased in DSS-treated *Trpa1*^+/+^ (DSS-*Trpa1*^+/+^) mice (b). In DSS-treated *Trpa1*^−/−^ (DSS-*Trpa1*^−/−*)*^ mice the withdrawal responses to forces of 0.008 g, 0.02 g, 0.04 g (*P* < 0.001 vs. DSS-*Trpa1*^+/+^), 0.07 g and 0.16 g (*P* < 0.01 vs. DSS-*Trpa1*^+/+^) were significantly smaller than in DSS-treated *Trpa1*^+/+^ mice (**b**). In the periorbital (facial) region, there was no DSS × genotype interaction and any DSS or genotype effect on the withdrawal threshold to mechanical stimulation under basal conditions (two-way ANOVA, **c**). Two-way ANOVA of the data obtained after DSS treatment (post DSS) disclosed a DSS × genotype interaction (*P* < 0.01), and post-hoc Bonferroni’s multiple comparison analysis demonstrated significant differences under post DSS conditions: *P < 0.05 vs. Con-*Trpa1*^+/+^; ^$^*P* < 0.05 vs. DSS-*Trpa1*^+/+^ (**b,c**).

In order to further elucidate the contribution of TRPA1 to somatic pain hypersensitivity in mice with DSS-induced colitis, we assessed the sensitivity of abdominal and periorbital regions to mechanical stimuli in *Trpa1*^+/+^ and *Trpa1*^−/−^ mice following DSS treatment. Under basal conditions, *Trpa1*^+/+^ and *Trpa1*^−/−^ mice did not differ in their withdrawal response in the abdominal region (Fig. 2a). Following treatment with DSS for 7 days, the abdominal withdrawal response of *Trpa1*^+/+^ mice was significantly enhanced relative to that of control *Trpa1*^+/+^ mice (Fig. 2b). In contrast, the abdominal withdrawal response of DSS-treated *Trpa1*^−/−^ mice remained unaltered relative to that of control *Trpa1*^−/−^ and control *Trpa1*^+/+^ mice but was significantly smaller than that of DSS-treated *Trpa1*^+/+^ mice (Fig. 2b). Two-way ANOVA disclosed a significant interaction between DSS and genotype at the test forces of 0.008 g, 0.02 g, 0.04 g, 0.07 g and 0.16 g. Post-hoc testing with Bonferroni’s multiple comparison analysis revealed that the withdrawal responses to these test forces were significantly increased in DSS-treated *Trpa1*^+/+^ mice (Fig. 2b), while the hypersensitivity to these test forces was absent in DSS-treated *Trpa1*^−/−^ mice (Fig. 2b).

Under basal conditions there was no difference between *Trpa1*^+/+^ and *Trpa1*^−/−^ mice in their withdrawal threshold to mechanical stimulation of the periorbital region between *Trpa1*^+/+^ and *Trpa1*^−/−^ mice (Fig. 2c). DSS treatment for 7 days lowered the withdrawal threshold in *Trpa1*^+/+^ mice to a significant extent (Fig. 2c). This colitis-related mechanical hypersensitivity of the periorbital region was absent in *Trpa1*^−/−^ mice (*P* < 0.01 by two-way ANOVA, Fig. 2c). Taken together, these data show that the mechanical hypersensitivity associated with DSS-induced colitis in both the abdominal and facial region is prevented by genetic deletion of TRPA1.

### DSS-induced colitis remained unaffected by the TRPA1 antagonist HC-030031 and by genetic deletion of TRPA1

**Figure 3:**
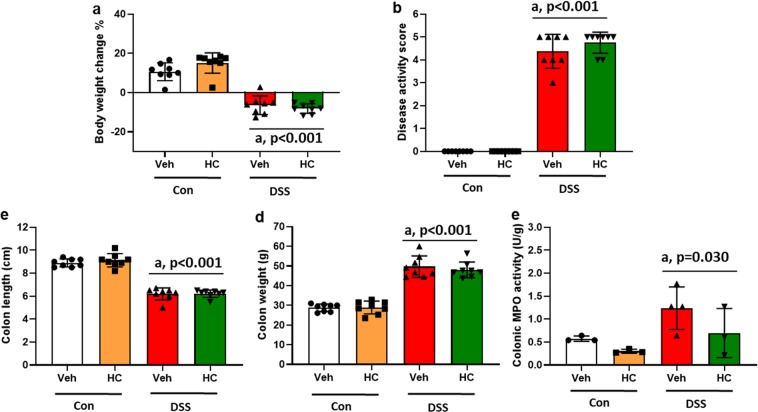
Effect of the selective TRPA1 antagonist HC-030031 (HC) or its vehicle (Veh) on colitis-related parameters (**a–e**) in control (Con) and DSS-treated mice. The parameters were measured on day 8 following a 7-day treatment period (study 1, set 1). HC (100 mg/kg, i.p.) or its vehicle was injected one hour prior to sacrifice of mice. The body weight change shown in panel A is calculated as a percentage of the weight measured before the treatment started. Values are expressed as means +/− SEM (n = 8 mice, A–D) (n = 3–4 mice, E). Two-way ANOVA did not show any DSS × HC interaction and any HC effect, but revealed a DSS effect (^a^*P* < 0.05 vs. Con).

In order to test whether the reduction of sensitivity to mechanical stimuli by blockade or deletion of TRPA1 is related to a change of DSS-induced colitis, we tested the effect of HC-030031 and TRPA1 deletion also on colitis-related parameters. Analysis of the readouts did not reveal any significant interaction between DSS and HC-030031 treatment. However, two-way ANOVA disclosed a main factor effect of DSS (*P* < 0.001) which caused a loss of body weight (Fig. 3a), increased disease activity score (Fig. 3b), reduced colon length (Fig. 3c), increased colon weight (Fig. 3d) and enhanced colonic MPO activity (P < 0.05) (Fig. 3e). Pharmacological blockade of TRPA1 with HC-030031 did not modify any of these parameters in control and DSS-treated mice to a significant extent (Fig. 3a–e). Taken together, these data show that DSS treatment for 7 days induced colitis which remained unaffected by TRPA1 blockade with HC-030031.

**Figure 4:**
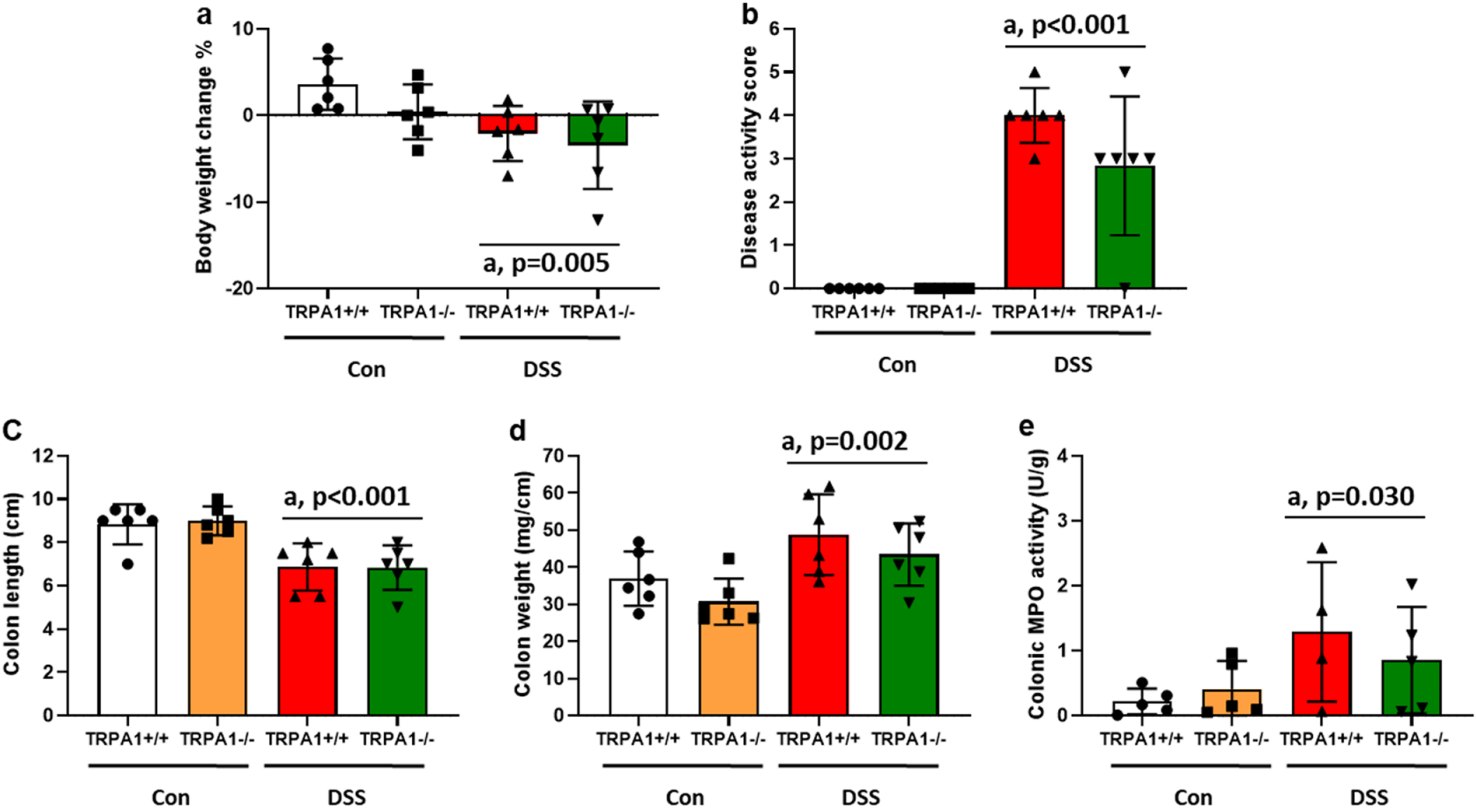
Effect of genetic deletion of TRPA1 on colitis-related parameters (**a–e**) in control (Con) and DSS-treated mice. The parameters were measured on day 8 following a 7-day treatment period (study 1, set 2). Values are expressed as means +/− SEM (n = 6 mice, A-D) (n = 5 mice, E). Two-way ANOVA did not show any interaction between DSS × genotype and any genetic deletion effect but revealed a DSS effect (^a^*P* < 0.05 vs. Con).

In order to further explore the possible contribution of TRPA1 to DSS-induced colitis, we tested the effect of genetic deletion of TRPA1 by examining the severity of colitis in *Trpa1*^+/+^ and *Trpa1*^−/−^ mice. Treatment of *Trpa1*^+/+^ mice with DSS for 7 days induced body weight loss (*P* < 0.01) (Fig. 4a), increased disease activity score (*P* < 0.001) (Fig. 4b), reduced colon length (*P* < 0.01) (Fig. 4c), increased colon weight (*P* < 0.001) (Fig. 4d) and enhanced colonic MPO activity (*P* < 0.05) (Fig. 4e). Genetic deletion of TRPA1 did not alter any of these parameters to a significant exrent (Fig. 4a–e). Thus, the present data exclude a role of TRPA1 in the colitis-related parameters under study.

### DSS treatment increased the relative expression of TRPA1 mRNA in DRGs and led to sensitization of DRG cells

**Figure 5:**
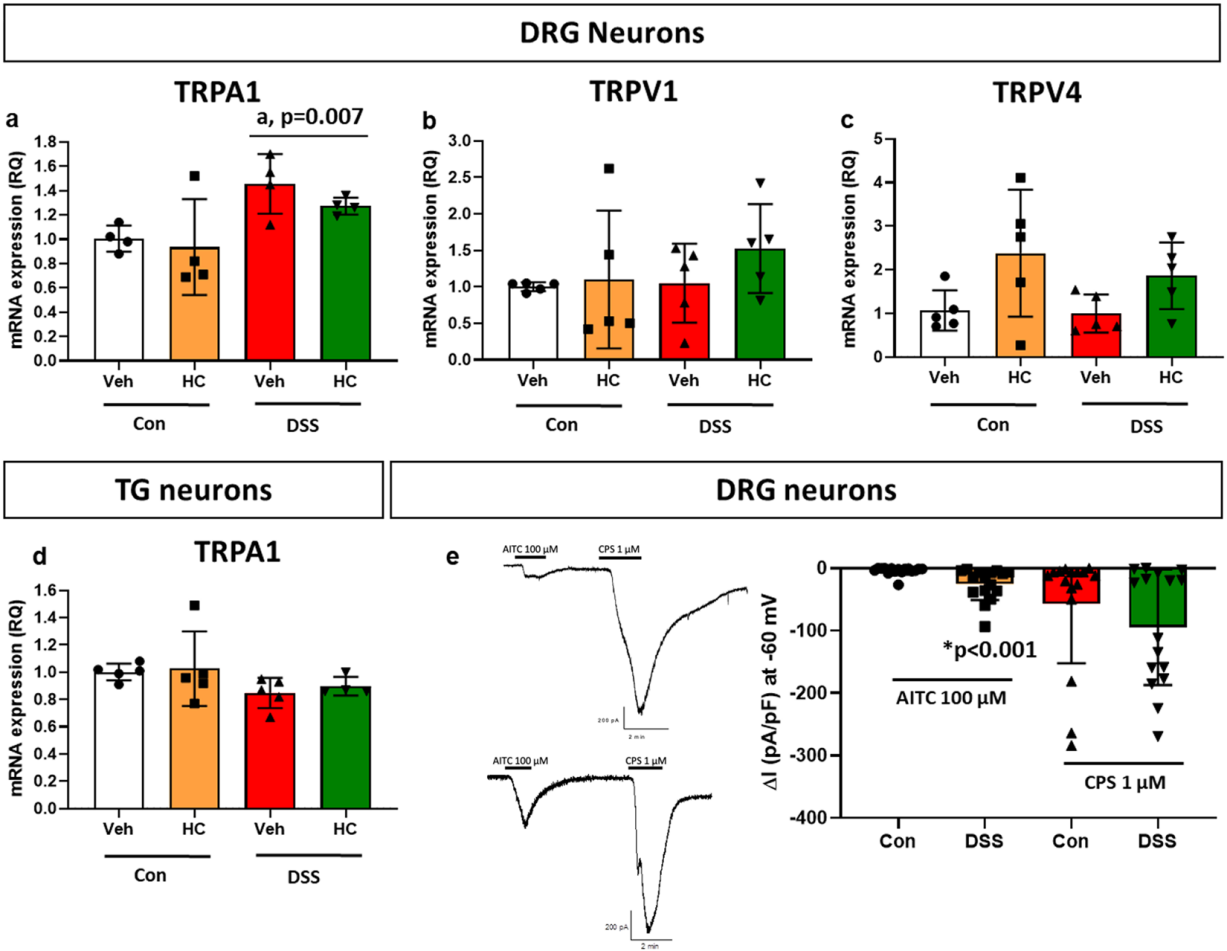
Effect of HC-030031 (HC) or its vehicle (Veh) on expression of TRPA1, TRPV1 and TRPV4 mRNA in lumbosacral DRGs (**a–c**) and TGs (**d**) isolated from control (Con) mice and mice treated with DSS for 7 days. HC (100 mg/kg, i.p.) or its vehicle was injected one hour prior to sacrifice of mice. Values are expressed as means + SEM (n = 4–5). Two-way ANOVA did not show any DSS × HC interaction and any HC effect but revealed a DSS effect on the expression of TRPA1 mRNA (^a^*P* < 0.05). (**e**) typical traces and pooled data of the inward currents evoked by AITC and capsaicin (CPS) in thoracosacral DRG neurons isolated from control (Con) mice and mice treated with DSS for 7 days. ΔI refers to the difference between current amplitude measured at -60 mV before and after application of AITC/CPS. The inward currents are shown as current density pA/pF. Values are expressed as means - SEM (n = 16 neurons from 3 mice/group). Mann-Whitney U test demonstrated a significant difference between the AITC responses of the Con and DSS groups (*P < 0.05).

In order to explore the effect of colitis on TRPA1 expression and sensitivity at the sensory neuron level, we examined the effects of DSS treatment on the relative expression of TRPA1 mRNA in lumbosacral DRGs and TGs (study 2, set 1) and on TRPA1 responsiveness of DRG neurons in culture (study 2, set 2). DSS treatment for 7 days increased the expression of TRPA1 mRNA in the DRGs (Fig. 5a) but not TGs (Fig. 5d) (DSS main factor effect, *P* < 0.01). In contrast, HC-030031 had no significant influence on TRPA1 mRNA expression in DRGs and TGs, and statistical analysis of the data showed that there was no significant interaction between the DSS and HC-030031 treatments (Fig. 5). The expression of TRPV1 and TRPV4 mRNA in DRG neurons was not altered by DSS treatment (Fig. 5b,c).

In the electrophysiologic studies, thoracosacral DRG neurons in culture were challenged with the TRPA1 agonist AITC or the TRPV1 agonist capsaicin, and peak inward currents were recorded, normalized to membrane capacitance and expressed as current density (pA/pF). Both AITC and capsaicin evoked inward currents at a voltage clamp of -60 mV (Fig. 5e). Inward currents induced by capsaicin were not significantly altered by DSS treatment (Fig. 5e). In contrast, the AITC response of DRG cells isolated from DSS-treated mice was significantly amplified when compared with that of DRGs from control mice (Fig. 5e). As the data were not normally distributed, analysis with a non-parametric test revealed significant differences in inward current (*P* < 0.001, Fig. 5e) between the DRG neurons from control and DSS-treated mice. These findings indicate that TRPA1 in DRG neurons is both upregulated and sensitized in mice with DSS-induced colitis.

## Discussion

Here we report that colonic inflammation evoked by DSS was associated with an increase in mechanical sensitivity observed both in the abdominal and periorbital region of the mouse. The presence of mechanical hyperalgesia in the periorbital region of mice with DSS-induced colitis, as reported here, is consistent with the mechanical hypersensitivity found in the abdominal and plantar skin reported previously^24^. The current data show that colitis evoked by DSS leads to widespread hypersensitivity to mechanical pain stimuli in somatic tissues. The increase in mechanical sensitivity in the abdominal as well as periorbital region and plantar skin indicates that colitis not only elicits referred hyperalgesia in dermatomes of overlapping visceral and somatic primary afferent neurons but also extends mechanical hypersensitivity beyond these dermatomes.

Our current data furthermore demonstrate that TRPA1 is an important signaling factor in colitis-associated hyperalgesia at the primary sensory neuron level because the severity of colitis was not affected by TRPA1 blockade or deletion. This ion channel is abundantly expressed in a subpopulation of DRG and TG sensory neurons and thought to be an important nocisensor ion channel, given that it is activated by mechanical and chemical pain stimuli^15,16,31^_._ The current findings underpin this contention as they show that TRPA1 plays an essential mediator role in the somatic mechanical hyperalgesia associated with DSS-induced colonic inflammation. This conclusion is based on two sets of experimental data. First, genetic deletion of TRPA1 prevented DSS-induced colitis from inducing mechanical hypersensitivity in both the abdominal and periorbital skin. Second, the selective TRPA1 antagonist HC-030031 inhibited the mechanical hypersensitivity that had developed after induction of colitis, and this observation was also made in both the abdominal and periorbital skin.

The implication of TRPA1 in colitis-associated somatic hypersensitivity is in line with a number of reports that attribute TRPA1 a role in visceral nociception and hyperalgesia. For instance, oral administration of HC-030031 (75 mg/kg) one hour prior to intracolonic AITC instillation inhibits the referred pain which the TRPA1 stimulant elicits in the abdominal skin^32^. Furthermore, the TRPA1 blocker TCS-5861528^33^ as well as intrathecal administration of TRPA1 antisense oligonucleotides^23^ attenuate mechanical hypersensitivity to colonic distension in trinitrobenzene sulfonic acid-induced colitis. It should be noted in *Trpa1*^−/−^ mice mechanical allodynia was inhibited at forces up to 0.16 g were, whereas the TRPA1 antagonist attenuated responses to the application of a lower force (0.04 g, Fig. 1b). However, the apparent discrepancy might be due to the relatively insufficient dose of the antagonist that could not achieve a complete channel inhibition. Nonetheless, our findings based on both pharmacologic inhibition and genetic deletion of TRPA1 corroborate the conclusion that TRPA1 is implicated in the somatic mechanical hyperalgesia that becomes manifest in response to colonic inflammation. This implication of TRPA1 is in keeping with the known roles of TRPA1 in nociception.

The widespread mechanical hypersensitivity that is elicited by colonic inflammation in somatic tissues extends beyond the boundaries of referred abdominal pain and thus is likely to involve central sensitization that would explain hyperalgesia in the periorbital and plantar skin. It was beyond the scope of this study to analyze the underlying pathways in their entirety, but the observations related to TRPA1 enable us to draw some relevant conclusions. One conclusion is that TRPA1 expressed by DRG neurons supplying the colon is the prime factor that initiates referred pain and central sensitization in response to colitis. This conclusion is consistent with the upregulation of TRPA1 in the inflamed mouse colon as well as in colon specimens obtained from UC and CD patients^34^). Furthermore, DSS-induced colitis led to upregulation of TRPA1 mRNA in DRGs but not TGs, which suggests that TRPA1-expressing trigeminal sensory neurons do not contribute to the mechanical hyperalgesia seen in the periorbital skin. We therefore argue that facial hypersensitivity associated with DSS-induced colitis is primarily due to central sensitization processes. Treatment with DSS for 10 days has been reported to increase the expression of TRPA1 and TRPV1 mRNA in DRGs (L6-S1), a finding that has been linked to the effect of colitis to cause anxiety- and depression-like behaviors associated with abdominal discomfort^35^. Intrigued by this report we found that the inward currents evoked by the TRPA1 agonist AITC were enhanced in cultured DRG neurons taken from DSS-treated mice, whereas responses to the TRPV1 agonist capsaicin remained unchanged. We therefore conclude that it is primarily the function of TRPA1 which, relative to TRPV1, is upregulated in DRG neurons under conditions of colitis, although the somatic vs. visceral projection of TRPA1-hyperresponsive DRG neurons awaits to be analyzed.

TRPA1-expressing primary sensory neurons contain the neuropeptides SP and CGRP which when released from their peripheral terminals mediate neurogenic inflammatory processes^15,16^. Apart from neurons, non-neuronal cells in the GI tract such as enteroendocrine cells also express TRPA1, are activated by colonic inflammation and contribute to the inflammatory process^15,36^. TRPA1 activation has been reported to protect from colitis by reducing the expression of several proinflammatory cytokines, chemokines and neuropeptides^34^. In the present study, however, we found that the TRPA1 antagonist HC-030031 as well as genetic deletion of TRPA1 failed to significantly alter the severity of DSS-induced colitis as judged by body weight, colon length, colon weight, colonic MPO activity and disease activity score. These findings confirm a previous report that acute treatment with HC-030031 fails to modify experimentally induced colitis in mice^25^. There is, however, conflicting evidence in the literature as to which extent TRPA1 inhibition or deletion impacts on experimental colitis. Thus, DSS treatment of TRPA1 knockout mice has been found to increase the disease activity score and to elevate proinflammatory cytokine and neuropeptide concentrations in the colon^34^ while other reports indicate that genetic deletion of TRPA1 has no effect^37^ or protects from experimentally induced colitis^29,38^. Here we confirm that pharmacologic blockade of TRPA1 with HC-030031 and genetic deletion of TRPA1 has no overt effect on the severity of DSS-induced colitis in mice. Whether the ability of TRPA1 inhibition and deletion to reduce colitis-associated somatic mechanical hypersensitivity is related to the observed nominal reduction of colonic MPO activity (Figs. 3 and 4) requires further investigation.

The current findings indicate that TRPA1 contributes to the somatic mechanical hypersensitivity that is induced by colonic inflammation. We assume that this role of TRPA1 takes place primarily at the primary sensory neuron level because the severity of colitis is not affected by TRPA1 blockade or deletion. Although upregulation of TRPA1 expression and sensitivity has been observed in DRG neurons such molecular changes should not be implicated in somatic pain sensitivity. In fact, mRNA levels were unchanged in TG despite that mechanical allodynia was detected in the periorbital area. Another possible interpretation may, however, be advanced. TRPA1 is expressed in neuropeptide containing sensory neurons^39,40^ and its activation results in SP and CGRP release. Sensory neuropeptides may evoke mechanical allodynia^41^ and sensory neuropeptides have been implicated in TNBs- and DSS-induced colitis^29^. Thus, following this previous evidence^29^ we advance the hypothesis that TRPA1 activation during DSS-induced colitis may release sensory neuropeptides which may contribute to peripheral and central sensitization resulting in mechanical allodynia. In addition, sensitization processes at higher levels of the central nervous system are proposed to account for the spread of somatic hyperalgesia beyond the dermatomes of overlapping visceral and somatic primary afferent neurons. These TRPA1-mediated processes may have relevance to some of the extraintestinal manifestations of IBD^10^.

## Materials and Methods

### Ethic statement

All studies were conducted at the Department of Health Sciences, Section of Clinical Pharmacology and Oncology, of the University of Florence. The *in vivo* experiments and tissue collections were carried out according to the Directive 2010/63/EU of the European Parliament and Council and Italian legislation (DL 26/201) for animal care procedures and under the University of Florence research permits #204/2012-B and #194/2015-PR. The experimental conditions and weight loss of mice were monitored daily. All studies involving animals were reported in accordance with the ARRIVE guidelines for reporting experiments involving animals^42,43^.

### Animals

C57BL/6 mice (male, 20-25 g, 8 weeks; Envigo), and littermate wild type (*Trpa1*^+/+^) and TRPA1-deficient (*Trpa1*^−/−^) mice (male, 25-30 g, 8 weeks), generated by heterozygotes on a C57BL/6 background (B6.129P-Trpa1^tm1Kykw/J^; Jackson Laboratories)^44^, were used. Animals were housed two per cage under controlled conditions of temperature (set point 21 °C), air humidity (set point 50%) and a 12 h light/dark cycle. Behavioral experiments were conducted in a quite temperature-controlled (20–22 °C) room between 9 a.m. and 5 p.m. by an operator blinded to genotype and drug treatment. Animals were euthanized with inhaled CO_2_ plus 10–50% O_2_. Given that the study involved pain, the number of animals per experimental group was kept to a minimum of n = 6–8^24^ and the experiments limited to male animals to minimize suffering.

### Materials

The following compounds were supplied as follows: dextran sulfate sodium (DSS) (MP Biomedicals); Trizol, Ham’s-F12, horse serum, fetal bovine serum, penicillin, streptomycin L-glutamine, mouse nerve growth factor, poly-L-lysine, laminin, collagenase type 1 A, papain AITC, capsaicin, Hank’s Balanced Salt Solution (Sigma Aldrich); iScript cDNA Synthesis kit, SsoAdvanced Universal Sybr Green Supermix (Bio-Rad) HC-030031,[2-(1,3-dimethyl-2,6-dioxo1,2,3,6-tetrahydro-7H-purin-7-yl)-N-(4-isopropylphenyl) acetamide] was synthetized as previously described^45^.

### Study design and experimental protocols

The project consisted of two studies. In each study (study 1 and 2), mice were randomly allocated to two or four different groups (https://www.randomizer.org/). DSS colitis was induced by adding 2% (w/v) DSS (molecular weight: 36,000–50,000) to the drinking water for 7 days. The body weight of the animals was measured one day before the start of any treatment and on day 8^24^. After completion of the 7-day DSS treatment, the animals were randomly assigned to one of the following experimental settings. In study 1, the effect of intraperitoneal (i.p.) injection of the TRPA1 antagonist HC-030031 (100 mg/kg), or its vehicle (4% dimethyl sulfoxide, DMSO, and 4% Tween 20 in 0.9% NaCl) and the effect of genetic deletion of TRPA1 on the severity of DSS-induced colitis and on somatic pain sensitivity in the abdominal and periorbital (facial) skin region were examined as follows:

Study 1, set 1. C57BL/6 mice were randomly allocated to four treatment groups: group I (control, vehicle for HC-030031), group II (control, HC-030031), group III (DSS treatment, vehicle for HC-030031), and group IV (DSS treatment, HC-030031). On day 8, the von Frey hair test of mechanical pain sensitivity was performed. HC-030031 or its vehicle was administered 1 hour before the von Frey hair test.

Study 1, set 2. *Trpa1*^+/+^ and *Trpa1*^−/−^ mice were randomly allocated to two treatment groups: group I (control) and group II (DSS treatment). On day 8, the von Frey hair test of mechanical sensitivity was performed.

In study 2, the effect of the TRPA1 antagonist HC-030031 (100 mg/kg, i.p.) on various molecular and functional parameters in DRGs and TGs taken from DSS-treated C57BL/6 mice was examined as follows:

Study 2, set 1. The effect of HC-030031 or its vehicle on TRPA1 mRNA expression in DRGs and TGs taken from DSS-treated mice was examined. The animals were randomly allocated to four treatment groups: group I (control, vehicle for HC-030031), group II (control, HC-030031), group III (DSS treatment, vehicle for HC-030031), and group IV (DSS treatment, HC-030031). Lumbosacral DRGs and TGs were isolated one hour after injection of HC-030031 or its vehicle and subjected to TRPA1 mRNA expression analysis.

Study 2, set 2. The effect of TRPA1 sensitization in primary cultures of DRGs taken for DSS-treated mice was investigated. Following treatment with DSS or vehicle for 7 days the animals were euthanized, and cultured DRGs were tested for their sensitivity to the TRPA1 agonist AITC and the TRPV1 agonist capsaicin by electrophysiology.

At the end of each study, the animals were euthanized, and the severity of colitis was assessed by recording of a disease activity score. In addition, colon length (without stretching), weight (after cleaning) and colonic MPO activity were estimated.

## Experimental Techniques and Readouts

### Disease activity score

The disease activity score based on appearance and stool consistency was recorded. Normal and abnormal appearance scored 0 and 1, respectively. Normal, soft but formed and loose stool scored 0, 1 and 2, respectively, and the absence of bleeding or the presence of blood traces or gross bleeding in the perianal region scored 0, 1 and 2, respectively. The absence or presence of blood in the stool was evaluated with the Hemdetect test (DIPROmed) and scored as 0 or 1. The sum of all scores in each category yielded scores of 0 to 6^24,46^.

### Colonic MPO activity

Tissues were weighed and homogenized in lysis buffer (80 mM sodium acetate buffer, pH 5.4, plus 0.5% hexadecyltrimethylammonium bromide, pH 7.4). Tissue debris was pelleted by centrifugation (two runs at 11,200 × g at 4 °C for 20 minutes). Supernatants were used for measurement of MPO activity as a marker of neutrophil infiltration. For assay, 10 µl of supernatant and 220 µl of 80 mM sodium acetate buffer (pH 5.4) were added in triplicate to a 96-well plate. The reaction was initiated by the addition of 20 µl of 18.4 mM tetramethylbenzidine. The mixture was incubated for 3 minutes at 37 °C and then immersed in an ice bath. The reaction was stopped by the addition of 30 µl of acetic acid (1 N), and the absorbance was monitored at a wavelength of 630 nm. All standard and sample values were subtracted with blank control values, and MPO activity was quantitated using a standard curve. The MPO activity was expressed as MPO units (U) per g of tissue^47^.

### von Frey hair test

The mechanical pain sensitivity of the abdominal and facial skin region was evaluated with von Frey filaments by methods described previously^24,41^. Briefly, for the abdominal mechanical hypersensitivity assessment, mice were habituated and trained to stay in small plexiglas compartments on a wire mesh floor for one hour. Subsequently von Frey filaments were applied to the abdomen (between diaphragm and genitals). The tests were performed by a trained blinded observer. In the test on the abdominal skin region, individual filaments were tested in an ascending order involving 0.008, 0.02, 0.04, 0.07, 0.16, 0.4 and 0.6 g forces. Each force was applied 10 times to the abdominal surface. The maximal duration of each force application was 2–5 seconds, and the inter-stimulus interval was 2–3 minutes. Following each challenge, the withdrawal response was quantified either as 1 (withdrawal of abdominal wall or paw, licking or retraction of animal) or 0 (no response). All counts in response to an individual filament were averaged. Withdrawal responses to low forces reflect high mechanical pain sensitivity^24^.

For periorbital mechanical allodynia, mice were allocated in a restraint apparatus consisting in an individual clear three-walled plexiglass box (4 H × 4 W × 10 L cm) with an opening for the tail and one for the head and front paws, located on a platform to allow the operator to access to the periorbital area. The box size allowed for head and forepaw movements but prevented the animal from turning around inside it. Periorbital mechanical allodynia was evaluated in the periorbital region over the rostral portion of the eye (*i.e*., the area of the periorbital region facing the sphenoidal rostrum) of the mice^41^. The individual filaments were tested in an ascending or descending order involving 0.008, 0.02, 0.04, 0.07, 0.16, 0.4, 0.6, 1, 1.4 g forces. The 0.16 g force was applied first to the periorbital region, and then the next filament was used based on the animal’s response. Following each challenge, the withdrawal response was quantified either as 1 (sharp withdrawal of face) or 0 (no response). In case of a sharp withdrawal of the face the next filament of lower intensity was used, while the next filament of higher intensity was used in case of no response. Subsequently, 5 more responses were recorded from each mouse in the same manner. The maximal duration of each force application was 2 second, and the inter-stimulus interval was 2-3 minutes. The withdrawal threshold was calculated according to the up-down method. Low withdrawal thresholds reflect high mechanical pain sensitivity^48–50^.

The mechanical sensitivity in the abdominal and periorbital region was assessed with the von Frey hair test under three experimental conditions (study 1, set 1): basal - prior to any treatment, pre-vehicle/HC-030031 - following control or DSS treatment but prior to injection of HC-030031 or its vehicle, and post-vehicle/HC - following control or DSS treatment and injection of HC-030031 or its vehicle.

### RT-PCR

RNA was extracted from the isolated DRGs and TGs using Trizol according to the manufacturer’s protocol. The RNA concentration and purity were assessed spectrophotometrically by measuring their absorbance at 260 nm and 280 nm. Aliquots of 100 ng RNA were reverse transcribed with the iScript cDNA Synthesis kit according to the manufacturer’s protocol. For relative quantitation of mRNA, real time PCR was performed on Rotor Gene Q (Qiagen). The specific primers used for amplification and quantitation of mRNA were as follows: 18S-FW (forward): 5′-CGCGGTTCTATTTTGTTGGT-3′, 18S-RE (reverse): 5′-AGTCGGCATCGTTTATGGTC-3′ (NCBI Ref Seq: NR_003278.3); TRPA1-FW: 5′-CAGGATGCTACGGTTTTTTCATTACT-3′, TRPA1-RE: 5′-GCATGTGTCAATGTTTGGTACTTCT-3′(NCBI Ref Seq: NM_177781.4). The chosen reference gene was the 18S. The SsoAdvanced Universal Sybr Green Supermix was used for amplification, and the cycling conditions were the following: samples were heated to 95 °C for 1 minute followed by 40 cycles of 95 °C for 10 seconds, and 65 °C for 20 seconds. PCR reaction was carried out in triplicate. Relative expression of TRPA1 mRNA was calculated using the 2^−Δ(ΔCT)^ comparative method, with each gene normalized against the internal endogenous reference 18S gene for the same sample^51^.

### Isolation and culture of primary sensory neurons

After euthanization of mice, the vertebral column was dissected from head to tail and cleaned with Hank’s Balanced Salt Solution (HBSS) containing penicillin and streptomycin (both, 100 U/ml). The ganglia were collected in HBSS and cleaned. Subsequently, ganglia were digested into HBSS containing collagenase type 1A (2 mg/ml) and papain (1 mg/ml) for 25 minutes at 37 °C. After being washed with HBSS, ganglia were pelleted and resuspended in Ham’s-F12 containing heat inactivated horse serum (HS, 10%), heat-inactivated fetal bovine serum (FBS, 10%), penicillin and streptomycin (both, 100 U/ml) and L-glutamine (2 mM). Ganglia were dissociated in single cells by several passages through a series of syringe needles (23–25 G) and centrifuged (11,000 rpm, 20–25 °C, 5 minutes). The neurons were then suspended in Ham’s-F12 containing HS (10%), FBS (10%), penicillin and streptomycin (both, 100 U/ml), L-glutamine (2 mM) and mouse nerve growth factor (100 ng/ml), and plated on glass coverslips coated with poly-L-lysine (8.3 µM) and laminin (5 µM)^52^. Neurons were cultured for 24 hours before being used in electrophysiological experiments^52^.

### Electrophysiology

For recording on isolated DRG neurons, a glass cover slip with neurons was transferred to a compartment on the stage of a microscope (Olympus CKX41). DRG neurons were superfused with a standard extracellular solution (10 mM HEPES, 10 mM D-glucose, 147 mM NaCl, 4 mM KCl, 1 mM MgCl_2_ and 5 mM CaCl_2_) which was maintained at a flow rate of 2 ml/min and pH 7.4 (adjusted with NaOH). A Sutter Instruments puller (model P-87) was used to pull and create borosilicate glass electrodes (Harvard Apparatus) with a final tip resistance of 4-7 mOhm. The pipette solution which was used for recording contained 120 mM CsCl, 3 mM Mg_2_ATP, 10 mM 1,2-bis(o-aminophenoxy) ethane-N,N,N′,N′-tetraacetic acid, and 10 mM 4-(2-hydroxyethyl)-1-piperazineethanesulfonic acid-Na adjusted to pH 7.4 with CsOH. The temperature was maintained at 20-22 °C throughout the experiment. Recordings were made by using an Axopatch 200B amplifier (Axon Instruments) and analyzed and stored with the pClamp 9.2 software (Axon Instruments). The neurons were voltage-clamped at a holding potential of −60 mV, and signals were sampled at 1 kHz and low-pass filtered at 10 kH. TRPA1 currents were detected as inward currents activated by AITC (100 µM). Capsaicin (1 µM) was used to identify capsaicin-sensitive nociceptive neurons. Consequently, the peak currents were recorded and normalized to membrane capacitance. The peak currents were averaged and expressed as a mean of the current density (pA/pF)^52^. Cell capacitance was used to estimate neuronal diameter by assuming an approximated spherical cell shape according to the calculated Cm for all biological membranes of 1 µF/cm^2^ and to the equation of the sphere surface: A = 4πr^2^ ^53^. The average cell diameter of DRG neurons included in the present research was 27.2 ± 1.8 µm corresponding to small-medium sized DRG neurons.

### Data and statistical analysis

The data and statistical analysis comply with the recommendations on experimental design and analysis in pharmacology^54^. The IBM SPSS 20, SigmaPlot 12.5 and GraphPadPrism 8.3.1 packages were used to analyze and plot the results. Before applying any statistics, the data were checked for normal distribution with the Shapiro-Wilk test. In study 1, set 1, DSS and treatment with HC-030031 were considered as the two main factors. In study 1, set 2, DSS and genetic deletion of TRPA1 were considered as the two main factors. Interactions between the two factors (two-way ANOVA) or three factors (repeated measures ANOVA) were considered significant if *P* ≤ 0.05. The post-hoc Bonferroni test was employed to compare differences among the treatment groups. When repeated measures ANOVA was applied, sphericity assumptions were checked by Mauchly’s test and, in case of violation of sphericity, the Greenhouse-Geisser correction was used. After repeated measures ANOVA, the independent samples t-test was used to compare two factors while post-hoc two-way ANOVA was used to test for differences and interactions between two factors (DSS and HC-030031). Comparisons between two groups were made with the independent samples t-test. In case of nonparametric distributions, the Kruskal-Wallis test was employed, followed by post-hoc testing with the Mann-Whitney U-test. The Bonferroni correction was used for pairwise comparisons^24^.
